# The predictors of food security and dietary diversity among internally displaced persons’ children (6–59 months) in Bamenda health district, Cameroon

**DOI:** 10.1186/s13031-023-00511-2

**Published:** 2023-03-23

**Authors:** Ayuk Betrand Tambe, Mbah Larissa Akeh, Nicholas Tendongfor, Thembekile Dhlamini, Given Chipili, Xikombiso Mbhenyane

**Affiliations:** 1 Department of Public Health and Hygiene, Faculty of Health Sciences, University of Buea, P.O. Box 063, Buea, Cameroon; 2 Division of Human Nutrition, Faculty of Medicine and Health Sciences, Stellenbosch University, Stellenbosch, South Africa; 3 Department of Nutritional Science, School of Applied Science and Technology, Mukuba University, P.O. Box 20382, Kitwe, Zambia

**Keywords:** Food insecurity, Dietary diversity, Associated factors, Internally displaced persons

## Abstract

**Background:**

Malnutrition remains a major cause of morbidity and mortality amongst children in displaced settings. Nutrition at this stage is crucial for the growth and development of the child. It is estimated that 41 million children under 5 years are obese/overweight, 159 million are stunted and 50 million are wasted worldwide. This study aimed to determine the prevalence and predictors of food insecurity and dietary diversity among internally displaced persons’ children from 6 to 59 months.

**Methodology:**

A cross sectional community-based study was conducted on 395 children aged 6–59 months from May 2021-June 2021. A multistage cluster sampling method was used to select the study participants. A validated structured questionnaire was used to collect data on sociodemographic characteristics, food security and dietary diversity. Predictors of food insecurity and dietary diversity were identified using logistic regression. The outputs were presented using adjusted odd ratio (AOR) with 95% confidence interval (CI).

**Results:**

The study results revealed that the level of household food insecurity was 91.6%, at risk of experiencing hunger (3.3%) and food secure (5.5%). Participants had mean dietary diversity score of 3.6 food groups, 51.6% had a low or inadequate dietary diversity and 48.4% had adequate dietary diversity. Children who were from households with monthly income of $101- $200 US dollars and had been displaced just once were 79% (AOR: 0.21, 95%CI: 0.07–0.60) and 84% (AOR: 0.16, 95%CI: 0.05–0.50) less likely to be food insecure compared to their counterparts respectively. While households with participants who Walked ≥ 10 min to fetch drinking water (AOR: 11.61 95%CI: 2.39–52.08) were more likely food insecure. In addition, household monthly income of ≥ $100 US dollars (AOR: 0.20, 95%CI: 0.07–0.56) had a reduced chance of providing low diversified food. Households that had received social grants (AOR: 2.15, 95%CI: 1.38–3.49) and walked ≥ 10 min to fetch drinking water (AOR: 2.43, 95%CI: 1.48–3.98) had a higher chance of providing low diversified food.

**Conclusion:**

Dietary diversity and household food insecurity was unacceptably low and high respectively among internally displaced children. Policymakers should prioritize strengthening both nutrition sensitive and specific activities that contribute to reduction of food insecurity and consumption of unbalance diet.

## Background of study

Malnutrition remains a global threat and one of the highest causes of morbidity and mortality in children under the age of five is malnutrition [1]. Malnutrition, directly or indirectly accounts for more than half of all children’s deaths, causing around 300,000 deaths each year [2]. Globally, in 2019, roughly 47 million children under 5 years of age were wasted, 14.3 million were seriously wasted and 144 million were stunted, while 38.3 million were overweight or obese [3]. WHO also reports that about 5.4 million children under five die per year in Sub-Saharan African countries, including Cameroon, with about 7 million deaths. According to Chiabi et al. 6 in every 100 Cameroonian children under five were malnourished [4] in 2012. However, substantial progress has been made as a recent study by Ngassa et al. revealed that about one in every three children under five were malnourished in Cameroon in 2022 [5].

There has also been good news on the global prevalence of child stunting which decreased by one-third between 2000 and 2019 [6]. However, more collective efforts is required to achieve the Sustainable Development Goals (SDG) 1, 2 and 3 (eliminate hunger, no poverty, good health and well-being) by 2030. One of the most important measure to address undernutrition is by achieving nutrition adequacy and increased production of food groups thus making the national diet balanced [7, 8]. Improvement can be made, but only by maintaining access not only to food for all people, but also to balanced foods that make up a healthy diet. Adverse consequences are likely to manifest themselves if the national diet are deficient in nutrient. However, limited data on the diversity of diet exit.

Several studies have reported that consequences of malnutrition include delay in physical growth and motor development, lower intellectual quotient (IQ), higher behavioral problems, poor social skills, and they are more susceptibility to contracting diseases. It may also lead to higher levels of chronic diseases in adulthood which may have an effect on the next generation, as malnourished females are more likely to give birth to low-weight babies [2, 9]. Malnutrition is not an easy equation for example simple problem equals to single and simple solution. There are various factors which are interrelated and together, are involved in causing malnutrition [10]. Some of the most common determinants are inadequate dietary intake and diseases caused by a set of underlying factors such as household food insecurity, poor maternal/child caring practices, lack of access to basic health services, lack of safe water supply and unhealthy living environment such as open defecation [11, 12]. However, these underlying causes themselves are influenced by economic, political, and sociocultural conditions; national and global contexts; capacity, resources, environmental conditions, and governance [13] as this is seen and experienced more in countries experiencing crisis.

Food security exists when all people have physical and economic access to enough, safe, and nutritious food at all times to suit their dietary needs and food choices in order to live an active and healthy life [14]. The three main components of food security are availability, accessibility and utilisation. Availability - there is sufficient supply of adequate food on hand [15]. Food may be produced in the home, imported commercially, or obtained through food aid [16]. Accessibility - sufficient income or other resources to access sufficient and suitable food through home production, purchasing, gathering, and other means. Food may be available, but it is inaccessible to those who lack sufficient land to cultivate or the financial means to purchase it [17]. Utilization - Appropriate food processing and storage procedures, adequate understanding and implementation of nutrition and child care concepts, and enough health and sanitation services ensure that food is used properly [18]. In addition, household food distribution amongst household members in relation to each person’s nutrient requirements is referred to as utilization. Biological use, which is associated with a person’s health, is also included in utilization [17].

In Cameroon, the political crisis occurring in the North West and South West since October 2016 has caused many families to be displaced and settled in neighboring safe cities (like regional headquarters), leaving behind their homes, their source of food and their source of income. In December 2018, World Food Programme declared an emergency response and developed a response plan to provide live saving humanitarian interventions in terms of General Food Assistance (GFA) to affected vulnerable displaced and local populations, which will progressively be expanded further to targeted nutrition support, school feeding and livelihood/resilience-promoting activities [18]. Before this intervention was carried out, an Emergency Food Security Assessment(EFSA) was done and it revealed that 50% of internal displaced persons (IDPs) in the North West were food insecure (52,080 people) with 13% being severely food insecure (13,650 people) while global acute malnutrition prevalence among children aged 6 to 59 months is in North West was 4.4% [18].

Despite the fact that there are non-governmental and humanitarian organizations carrying out intervention programs to help reduce the rate of malnutrition among internally displaced persons living in Bamenda with supervisory support from the government, malnutrition and food insecurity prevalence updates remain unknown. Hence, this study aim to report the prevalence of malnutrition in internally displace children (6-59months) in Bamenda Health District. In addition, determine the level of food insecurity, dietary diversity and identify the factors associated with food security and dietary diversity in internally displaced persons living in Bamenda Health District.

## Methods

### Study design

The study was a descriptive cross-sectional community-based study. The study was conducted in Bamenda Health District which is one of the 19 Health Districts in the North West Region in Cameroon. It is located at the heart of the North West Regional headquarter with a population of about 429,419 of 2019. As of October 2018, United Nations Office for the Coordination of Humanitarian Affairs (OCHA) estimated there were 105,000 IDPs in Northwest Region with 43% of this estimated population are children. The district comprises of 18 health facilities, 14 public (government owned health facilities) and 4 conventional (faith-based health facility). Despite that there are many other private health facilities operating in anonymity there is equally a practice of modern traditional medicine in line with the Cameroon national health policy which recognizes the role traditional medicine in the health domain of the country.

### Study participants

The sample size was estimated at 373 using the formula n = Z^2^×P(1-P) /d^2^, where: n = minimum sample size, z = confidence value = 1.96 for a 95% confidence interval, p = estimated prevalence of childhood malnutrition from a study done in a similar setting = 41.2% [19] and 5% of precision with an additional 22 participants for attrition. Due to the ongoing crisis, we were unable to access all the health areas and so convenient sampling was used to select 4 health areas and 15 communities (quarters) in these health areas. This was done for security reasons. The inclusion criteria were children between 6 and 59 months with at least one primary caregiver or adult above 21 years who could give information of the child and the household and could also provide consent for the child to be recruited into the study.

### Materials

The survey used validated questionnaires for data collection. To guarantee the validity of the questionnaire, a pilot study with 20 participants was carried to ensure the questions were clear, well formulated, appropriate for the study, suitable to use as a research tool and in line with the expectations of the study. General information about the households, demographic and socioeconomic factors, household food insecurity, 24-hour recall (dietary-intake) and dietary diversity were included in the questionnaire.

The food security and dietary diversity of children were evaluated using standard tools [20, 21]. Household food insecurity status was categorized into three categories (secured, moderate or at risk of and hunger) as recommended by the Food and Nutrition Technical Assistance (FANTA) guide [21]. The minimum acceptable dietary diversity for children 6–59 months was defined as consuming food from four or more of the standardized set of seven food groups on the preceding day of the study [20].

### Data collection procedures

Data collection ran from April 2021 to June 2021. Data collection was done by the research team with the help of a community mobilizers who help IDP households. The recruitment of IDPs started with the introduction to the community leader (locally refer to as quarter head), where the research team explained the purpose of the research. Each community mobilizer was assigned by his/her community leader to help recruit IDPs’ households. For those who agreed to participate in the study, the consent form was presented to them. Participants were also informed that participation was voluntary. Once the participants were enrolled and the consent gotten from the caregiver, the questionnaires were administered to the caregiver. For reliability, the questionnaires were checked daily for completeness, consistency and clarity. The results of the pilot study were not merged with those of the main study.

**Data management and dataanalysis**.

Data was checked manually for completeness then it was coded and entered into Microsoft Excel and it was later exported to SPSS version 26.0 for analysis. Exploratory data analysis was done to check missing values and outliers. Bivariate analyses was done using Chi square test to determine independent factors that were significantly associated with household food insecurity and dietary diversity. All variables with a *p* value less than or equal to 0.2 were entered into logistic regression to determine the risk factors. The statistical significance was declared at *p* value less than 0.05. The results were reported using adjusted odds ratio (AOR) with their 95% confidence interval.

## Results

### Sociodemographic characteristics of caregivers

A total of 395 participants were included in the study. The sample distribution of caregivers were male (3.3%) and female (96.7%). Majority of the caregivers were Christians (86.3%), literate (64.3%), 47.9% were employed and 87.8% earned a monthly income of less than 50, 000 FCFA ($100 USD). In addition, 48.9% of the caregivers received social grant from either private (25.3%) or non-governmental organization (23.5%) and household family sizes ranged between 2 and 16 persons with an average of 6.7 ± 2.3, persons. More than half (57.5%) of the caregivers walked for more than 10 min for fetch drinking water (Table 1).

**Table 1:** Socio-demographic characteristics of the study sample

Characteristic	Frequency	Percentage
Religion		
Christian	341	86.3
Muslim	54	13.7
**Level of education of the head of the household**		
Illiterate	141	35.7
Literate	254	64.3
**Occupational status**		
Employed	31	7.9
Self-employed	189	47.9
Unemployed	175	44.3
**Monthly income**		
< 50,000 ($100 USD)	347	87.9
50,001-100,000($101-$200 USD)	45	11.4
> 100,000 (>$200 USD)	3	0.8
**Received social grants**		
No	202	51.1
Yes	193	48.9
**Household size**		
≤ 5 persons	249	63.0
> 5 persons	146	37.0
**Time walked to fetch water**		
< 10 minutes (low risk)	168	42.5
≥ 10 minutes (high risk)	227	57.5
**Number of forced displacements**		
One time	222	56.2
More than one time	173	43.8
**Age group of the child in the household**		
6–24 months	117	29.6
25–60 months	278	70.4
**Some diseases the child suffered from at least once a month**		
Catarrh	79	20.0
Diarrhoea	203	51.4
**Deworming status**		
**None**	48	12.2
**Every 3 months**	196	49.6
**Every 6 months**	151	38.2
**Vaccination status**		
Complete vaccination dosage	260	65.8
Incomplete vaccination dosage	135	34.2
**Gender of the child**		
Female	161	40.8
Male	234	59.2

### Characteristics of the surveyed children

They were 395 children included in the study 59.2% were male and 40.8% were female with ages ranging from 6 to 59 months with an average of 38.4 ± 17.7 months. Most of the children (77.2%) had a good appetite and few had low appetite as a result of illness or not enough food available. About 65.8% of the children had received their complete vaccination dosage (vaccines recommended by World health organization for their various ages) while 34.2% did not receive complete vaccination. A few of the children (20.0%) were suffering from catarrh, 51.4% of the children had diarrhea at least once a month. About half 49.6% of the children received worm medicine every 3 months (Table 1).

### Feeding practices of the children

#### Household food insecurity

Food availability and accessibility was assessed using the Household Food Insecurity Access Scale (HFIAS). Majority (92.4%) of the household residents had to eat a smaller variety of food due to a lack of money to buy food. Less than two-third of the households members (72.7%) had no food to eat at homes and 81.8% had at least one household member who sleep hungry at night due to lack of food. Furthermore, 61.5% households had members who sometimes went a whole day and night without food due to lack of resources.

**Table 2:** Household food insecurity scalequestions

Household food insecurity scale questions During the past 30 days:	Frequency (n)	Percentage (%)
Households which did not have enough food	365	92.4
Households whose members were unable to eat the types of food they like more	368	93.2
Households whose member ate a small variety of food	373	94.4
Households whose members ate food they normally do not to eat	373	94.4
Household whose member ate smaller meals than they need	373	94.0
Household whose members ate lesser meals in a day	369	93.4
Households were at some point, there was no food to eat due to lack of resources	287	72.7
Household in which a member sometimes went to sleep hungry because of a lack of food	323	81.8
Households in which a member stayed the whole day and night without eating anything because there was no food	243	61.5

**Fig. 1:**
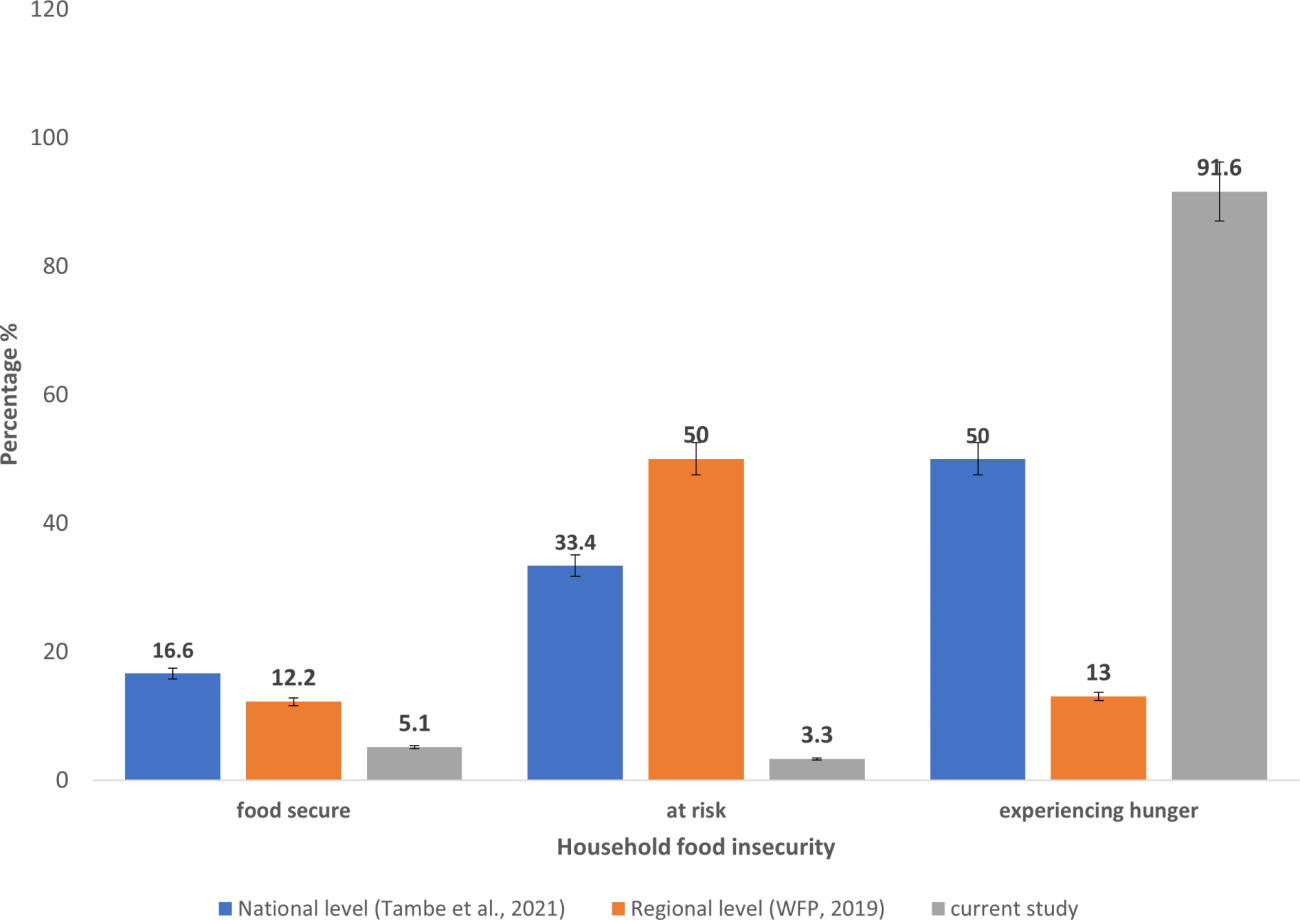
Household food security among surveyed household (n = 395)

Household hunger was also determined as displayed in Figs. 1 and 5.1% of households included in the study were classified as food secure, 3.3% were at risk of hunger and 91.6% experienced hunger. The study results were compared to the results of the region, as well as the national household hunger score (Fig. 1).

Multivariate logistic regression showed that households with monthly income of 50,001-100, 000 FCFA ( $101- $200 US dollars) (aOR: 0.21, 95%CI: 0.07–0.60, p = 0.0035), households with more than five persons (aOR: 0.31, 95%CI: 0.11–0.90, p < 0.032) and households with participants who walked ≥ 10 min to fetch drinking water (aOR: 11.61 95%CI: 2.39–52.08, p < 0.0021) were more likely food insecure. Furthermore, households that have been displaced just once (aOR: 0.16, 95%CI: 0.05–0.50, p < 0.0017) were less likely to be food insecure that their household counterparts that have been displaced two or more times (Table 3).

**Table 3:** Determinants of household food insecurity among households of internally displaced children of 6-59months in Bamenda Health District (n = 395)

			Univariate analysis		Multivariate analysis	
Characteristic	N	HFI (%)	Crude OR (95%CI)	P value	aOR (95%CI)	p value
**Religion**						
Christian	341	95.3	1			
Muslim	54	92.6	0.62(0.20;1.91)	0.402		
**Level of education of the head of the household**						
Illiterate	141	97.1	1		1	
Literate	254	93.7	0.43(0.14;1.33)	0.143	0.81(0.24;2.79)	0.743
**Occupational status of the head of the family**						
Employed	31	87.1	1		1	
Self-employed	189	95.8	3.35(0.94;11.89)	0.061	0.48(0.09;2.43)	0.373
Unemployed	175	95.4	3.09(0.87;10.98)	0.081	0.34(0.06;1.80)	0.206
**Monthly income (FCFA)** < 50,000 ($100 USD)	347	97.1	1		1	
50,001-100,000($101-$200 USD)	45	82.2	0.137(0.05;0.37)	**< 0.001**	0.21(0.07;0.60)	**0.0035**
> 100,000 (>$200 USD)	3	33.3	0.015(0.00;0.18)	**< 0.001**	0.05(0.01;0.68)	**0.0242**
**Received social grants**						
No	202	95.0	1			
Yes	193	94.8	0.95(0.39;2.34)	0.917		
**Household size**						
> 5 persons	249	96.0	1		1	
≤ 5 persons	146	93.2	0.57(0.23;1.40)	0.220	0.31(0.11;0.90)	**0.032**
**Time walked to fetch water**						
< 10 minutes (low risk)	168	89.3	1		1	
≥ 10 minutes (high risk)	225	99.1	1.20(1.07;1.34)	**< 0.001**	11.61(2.39;52.08)	**0.0021**
**Frequency of force displacement**						
More one time	222	98.2	1		1	
one time	173	90.8	0.18(0.06;0.55	**0.0026**	0.16(0.05;0.50)	**0.0017**
**Age group of the child**						
6–24 months	117	89.7	0.26(0.10;0.65)	**0.004**	0.26(0.10;0.69)	**0.006**
25–60 months	278	97.1	1		1	
**Dietary diversity Score**						
< 4 food groups	114	96.5	1			
≥ 4 food groups	281	94.3	0.60(0.20;1.84)	0.375		

#### Dietary diversity

All the mothers (100%) gave their children colostrum after birth. More than half 51.3% of the caregivers, introduced supplementary diet at the end of 6 months, meaning they carried exclusive breastfeeding for 6 months. All of the women agreed that colostrum is good for the baby and they gave breast milk to their children anytime they wanted it. About 53.9% did not give breast milk to their children for more than 12months. More than half 57.2% of the children, ate 3 to 4 times a day. Majority of the children (96.8%) ate meat or fish at least once a week while 95.6% ate fruits or vegetables at least once a week.

### Food groups consumed by the children

**Fig. 2:**
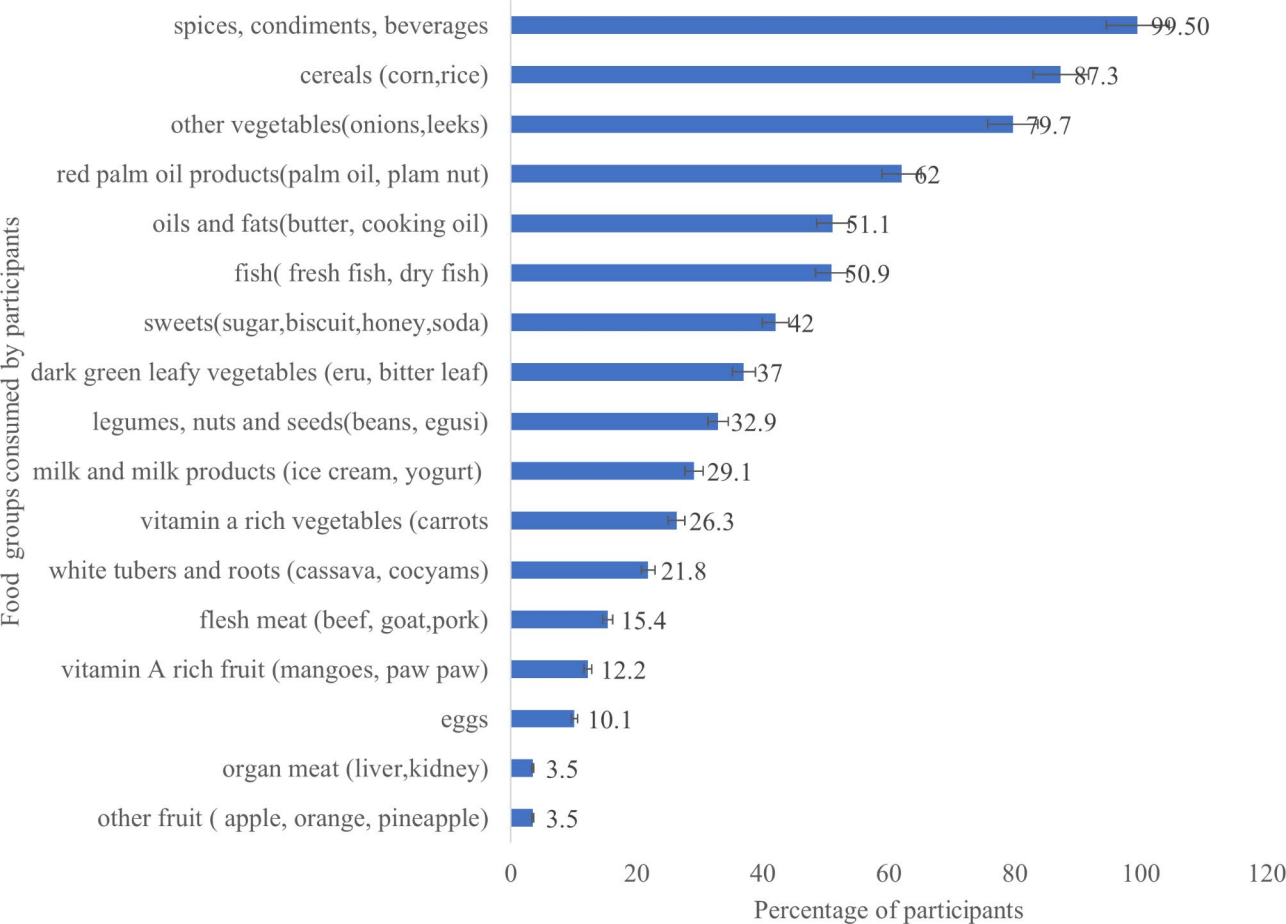
Food type consumed by internally displaced persons’ children aged 6–59 months (n = 395)

According to Fig. 2, the most food groups consumed by the children were spices and condiments, beverages (99.5%), cereals (87.3%), other vegetables (79.7%) and red palm products (62.0%) while the least consumed food groups were organ meat (3.5%) other fruits (3.5%), eggs (10.1%) and vitamin A rich fruits (12.2%). Only 1% of participants had an adequate DDS (a score of 12–13), 25.6% had a medium score (a score of 8–11), 71.9% had a low score (a score of 4–7) and 1.5% had a poor DDS (score of less than 3).

### Child dietary diversity score (CDDS)

The 17 food groups were collapsed into 7 food groups as per WHO classification of Child Dietary Diversity Score (CDDS). A score of four or more out of seven signifies adequate dietary diversity while a score below four signifies a low dietary diversity as per WHO guidelines. Current findings revealed that 51.6% of children had a low CDDS while 48.4% had an adequate CDDS.

### Micronutrients of interest and corresponding food groups consumed by children

The Table 4 shows the number of children who consumed food groups rich in vitamin A and iron. Less than two-thirds (62.0%) of the children consumed red palm oil and red palm oil products and 36.7% consumed dark green leafy vegetables. Only about 29.1% of the participants consumed milk. About half of the participant (50.6%) consumed fish, less than one-third (15.4%) consumed flesh meat while only 3.5% consumed organ meat.

**Table 4:** Micronutrients of interest and corresponding food groups in the dietary diversity questionnaire(n = 395)

Micronutrients	Food groups	Percentages
Vitamin A	Red palm oil and red palm oil products	62.0
	Dark green leafy vegetables	36.7
	Milk	29.1
	Vitamin A rich vegetables and tuber	26.3
	Vitamin A rich fruits	12.3
	Eggs	10.1
	Organ meat	3.5
Iron	Fish	50.6
	Flesh meat	15.4
	Organ meat	3.5

### Classification of dietary diversity at different dietary diversity levels

The different food groups consumed by more than ≥ 50% of the children at the different dietary levels was also evaluated. Most of the food consumed by people with low dietary diversity were cereals, other fruits and vegetables. The food groups consumed by children with medium dietary diversity were cereals, vitamin A rich fruits and dark vegetables, other fruits and vegetables, meat and or fish. Food groups consumed by people with high dietary diversity were cereals, vitamin A rich fruits and dark vegetables, meat and or fish, legumes, nuts and seeds, milk and milk products (Table 5).

**Table 5:** Food group consumed by ≥ 50% of households by dietarydiversity

lowest dietary diversity (≤ 3food groups)	Medium dietary diversity (4 and 5 food groups)	high dietary diversity (≥ 6 food groups)
cereals	cereals	cereals
other fruits and vegetables	vitamin A rich fruits and dark vegetables	vitamin A rich fruits and dark vegetables
	other fruits and vegetables	other fruits and vegetables
	meat/fish	meat/fish
		legumes, nuts and seeds
		milk and milk products

Multivariate logistic regression analyses compared with low dietary diversity score after adjusting for all potential confounders revealed that, children living in households with monthly income of ≥ 50, 000 FCFA ( $100 US dollars) (aOR: 0.20, 95%CI: 0.07–0.56, p = 0.0075) were less likely to) were more likely to have a child who did not meet minimum dietary requirements. One the other hand, children whose households had received social grants (aOR: 2.15, 95%CI: 1.38–3.49, p < 0.0008) and caregivers walked ≥ 10 min to fetch drinking water (aOR: 2.43, 95%CI: 1.48–3.98, p < 0.0004) were more likely to have a child who did not meet minimum dietary requirements (Table 6).

**Table 6:** Predictor of low dietary diversity among internally displaced persons’ children of 6–59 months in Bamenda health District (n = 395)

			Bivariate analysis		Multivariate analysis	
Characteristic	n	DDS < 4 (%)	Crude OR (95%CI)	p value	aOR (95%CI)	p value
**Religion**						
Christain	341	29.3	1			
Muslim	54	25.9	0.84(0.44;1.62)	0.609		
**Level of education of the head of the household**						
Illiterate	141	36.2	1		1	
Literate	254	28.9	0.58(0.37–0.91)	0.017	0.70(0.44–1.13)	0.1437
**Monthly income (FCFA)**						
< 50,000	347	31.7	1		1	
≥ 50,00	48	8.3	0.20(0.07–0.56)	0.0023	0.23(0.08–0.68)	0.0075
**Received social grants**						
No	202	21.3	1		1	
Yes	193	36.8	2.15(1.38–3.38)	0.0008	2.20(1.39–3.49)	0.0008
**Household size**						
≤ 5 persons	146	29.5	1.05(0.67–1.64)	0.8422		
> 5 persons	249	28.5	1			
**Time walked to fetch water**						
< 10 minutes (low risk)	168	17.9	1		1	
≥ 10 minutes (high risk)	225	37.0	2.70(1.68–4.36)	< 0.0001	2.43(1.48–3.98)	0.0004
**Frequency of forced displacement**						
More one time	222	32.0	1		1	
one time	173	24.9	0.70(0.45–1.10)	0.1217	0.83(0.52–1.33)	0.4381
**Age group of the child**						
6–24 months	117	26.5	0.85(0.52–1.37)	0.5012		
25–60 months	278	29.9	1			
**Sex of the child**						
Female	161	28.6	1			
Male	234	29.1	1.02(0.66–1.60)	0.9162		

## Discussion

The current study reported the prevalence and predictors of food insecurity and dietary diversity among internally IDPs’ children from 6 to 59 months Bamenda Health District, Cameroon. This study highlighted that 91.6% of households were food insecure and 51.6% had inadequate dietary diversity. Food security was mainly influenced by low monthly income, high frequency of forced displacements and long distance Walked by caregiver to fetch drinking water. While low dietary diversity was influenced by low household monthly income, receiving social grants and long distance Walked by caregiver to fetch drinking water.

**T**he present study reported that most of caregivers were female, literate and had low month income. Better education means more knowledge and a higher probability to earn more, proper management of resources, practice better health promoting behaviors specifically better food choices, and might develop better children centered caring practices. This study findings is in line with preceding literature evidence which revealed that the level of education of the caregivers was considered as major predictors of malnutrition, increasing the risk of undernutrition due to illiterate parents [22]. In this study, majority (87.8%) of the households had a monthly income less than one hundred United State dollars which might not be enough to ensure livelihood of the average of seven persons per household reported in the study area. An estimated average cost of food per month in Cameroon for one person ranges from $50 to $100, depending on age. Increase in household sizes might lead to decrease in the availability of food in the household. A study conducted by World Food Program in the North West region of Cameroon, showed that 13% of IDP households engaged in unskilled waged labour while 8% living off small business. Some 25% of IDP households did not have access to any income source, 8% rely on assistance from friends or 6% from begging and average household size of IDPs living in North West is 6 (World Food programme 2019) [18].

According to findings of this study, most of the participants (91.6%) were food insecure that is, they were experiencing hunger, a few participants (3.3%) were at risk of experiencing hunger while 5.1% were food secure. Food insecurity increases the chances of malnutrition occurring. This is because, there is not enough food (calories and nutrients) thereby increasing the risk of unhealthy eating. The prevalence of food insecurity from this study was higher when compared with the prevalence of 13% of a carried out in January 2019 by World Food Programme to assess household food security in North West Region [18]. In another study conducted in Cameroon by Tambe et al. revealed that 50% of the participants were experiencing hunger, these results were also lower when compared to the current study findings [23]. However, a study carried out in Sekela District, Western Ethopia, by Mulu et al. showed the prevalence of household food insecurity to be 74.1% [24]. A possible explanation for the high prevalence of household food insecurity could be linked to the fact that more than half of the household did not receive any form of social grants thus they were not part of any present intervention program. Another reason could be as a result of the deteriorating humanitarian crisis situation where more people have moved from the villages to the town, thereby increasing family sizes. Also, during the study period, the main roads linking Bamenda to neighboring villages which supply food were blocked. In a house where household income is low and large family size, there are higher chances of the house being food insecure, because there is not enough financial resources to ensure that the dietary needs of all the members of the household are met.

Further analysis showed that food security was mainly influenced by low monthly income, high frequency of forced displacement and distance walked to fetch drinking water. Current findings suggest that households with two or more forced displacement times were more likely to be food insecure as compared to their counterparts who were displaced just once. Majority of households in Cameroon solely depend on small scale farming for their livelihoods and support systems. Therefore, forced displacement disconnects them from these farms. Thus, forced displacement limits the household ability to generate income and may cause significant welfare losses [25]. Reconstruction for many communities and socio-economic recovery for majority of households might have to be preceded by food security interventions [26].

The dietary diversity assessment indicated the four major classes of food consumed by the children were; spices and condiments, beverages, cereals, other vegetables and red palm products. While the least consumed food groups were organ meat, other fruits, eggs and vitamin A rich fruits. The mean child dietary diversity was 3.6 food groups and more than half of the children (51.6%) had a low dietary diversity score of less than four. Meaning they consumed less variety and classes of food than the recommended food groups by WHO in the past 24-hours. Dietary diversity provides information on household access to food varieties, the eating patterns and behaviours of children within the household. These findings are different compared to a similar study conducted by Di Marcantonio et al. among IDP camps in Somali in 2019 which had a less (15%) of low child dietary diversity [27]. Due to low income, the caregivers are not able to provide a variety of foods to the children.

Low household monthly income, receiving social grants and caregiver trekking ≥ 10 min to fetch drinking water were independently associated with low dietary diversity among children 6–59 months. The current study showed that less than half of the participants do receive social grants from government institutions, non-governmental organization or private (family members or friends). Social grants such as food aids is a primary source of developmental assistance and mostly given to the highly affected household. It is thus important in estimating the level of food diversity indirectly caused by such shocks [26]. The findings of this study are differs with what Devereux et al. in Eastern Cape Province in South Africa found, which indicated that, caregivers received a social grant for at least one child. According to Devereux and Waidler, social grants were introduced about 21 years ago and improvements have been seen in food consumption, dietary diversity and food insecurity since 1998, and this has led to an improvement in acute malnutrition but declined in chronic malnutrition [28]. This difference could be explained by the fact that the amount and type of grants received by households are insufficient to meet the recommended dietary diversity.

### Limitations of the study

The current study had its own limitation which should be noted. Due to security reasons, data collection was limited to particular quarters in some selected health areas which were mostly urban areas. The use of 24-hour dietary recall questionnaire can under or overestimate the dietary intake as it is dependent on the caregivers’ ability to recall their children dietary intake and firmness of the interviewer. The single 24-hour dietary recall and HFIAS techniques used in this study does not reflect quantity and seasonal variation of dietary intake. In addition, the influence of the long-covid-19 context and cultural factors on food security and dietary diversity were not investigated in the current study. As a strength, appropriate sample size was used which allows for findings to be generalized across the entire study population and confounders were accounted for during analysis of predictors.

## Conclusions

Dietary diversity and household food insecurity was unacceptably low and high respectively among internally displaced children. Food security was independently associated with low monthly income, high frequency of forced displacement and distance walked by caregivers to fetch drinking water. While low household monthly income, receiving social grants and long distance walked by caregivers to fetch drinking water were predictors of low dietary diversity. Policymakers should consider strengthening both nutrition sensitive and specific interventions that contribute to reduction of food insecurity and consumption of unbalance diet.

## Data Availability

All raw materials are available and stored in the Department of Public Health and Hygiene, Faculty of Health Sciences, University of Buea-Cameroon and can be available upon reason request.
